# Dependence of post-segregational killing mediated by Type II restriction–modification systems on the lifetime of restriction endonuclease effective activity

**DOI:** 10.1128/mbio.01408-24

**Published:** 2024-07-09

**Authors:** Svetlana Kozlova, Natalia Morozova, Yaroslav Ispolatov, Konstantin Severinov

**Affiliations:** 1 Skolkovo Institute of Science and Technology, Center for Molecular and Cellular Biology, Moscow, Russia; 2 Peter the Great St. Petersburg Polytechnic University, St. Petersburg, Russia; 3 Physics Department, University of Santiago of Chile, Center for Interdisciplinary Research in Astrophysics and Space Science, Santiago, Chile; 4 Waksman Institute for Microbiology and Department of Molecular Biology and Biochemistry, Rutgers, State University of New Jersey, Piscataway, New Jersey, USA; 5 Institute of Gene Biology, Moscow, Russia; The Pennsylvania State University, University Park, Pennsylvania, USA

**Keywords:** restriction–modification, *Escherichia coli*, plasmid stability, post-segregational killing

## Abstract

Plasmid-borne Type II restriction–modification (RM) systems mediate post-segregational killing (PSK). PSK is thought to be caused by the dilution of restriction and modification enzymes during cell division, resulting in accumulation of unmethylated DNA recognition sites and their cleavage by restriction endonucleases. PSK is the likely reason for stabilization of plasmids carrying RM systems in the absence of selection for plasmid maintenance. In this study, we developed a CRISPR interference-based method to eliminate RM-carrying plasmids and study PSK-related phenomena with minimal perturbation to the *Escherichia coli* host. Plasmids carrying the EcoRV, Eco29kI, and EcoRI RM systems were highly stable, and their loss resulted in SOS response and PSK. In contrast, plasmids carrying the Esp1396I system were poorly stabilized; their loss led to a temporary cessation of growth, followed by full recovery. We demonstrate that this unusual behavior is due to a limited lifetime of the Esp1396I restriction endonuclease activity, which, upon Esp1396I plasmid loss, disappears approximately after two cycles of cell division, i.e., before unmethylated sites appear in significant numbers. Our results indicate that whenever PSK induced by a loss of RM systems, and, possibly, other toxin–antitoxin systems, is considered, the lifetimes of individual system components and the growth rate of host cells shall be taken in account. Mathematical modeling shows, that unlike the situation with classical toxin–antitoxin systems, RM system-mediated PSK is possible when the lifetimes of restriction endonuclease and methyltransferase activities are similar, as long as the toxic restriction endonuclease activity persists for more than two chromosome replication cycles.

**IMPORTANCE:**

It is widely accepted that many Type II restriction–modification (RM) systems mediate post-segregational killing (PSK) if plasmids that encode them are lost. In this study, we harnessed an inducible CRISPR-Cas system to remove RM plasmids from *Escherichia coli* cells to study PSK while minimally perturbing cell physiology. We demonstrate that PSK depends on restriction endonuclease activity lifetime and is not observed when it is less than two replication cycles. We present a mathematical model that explains experimental data and shows that unlike the case of toxin–antitoxin-mediated PSK, the loss of an RM system induced PSK even when the RM enzymes have identical lifetimes.

## INTRODUCTION

Stable inheritance of plasmids in bacterial populations can be mediated by “addiction modules,” which act through post-segregational killing (PSK) of cells that lose plasmids that carry them (1). Well-known examples of plasmid-borne addiction modules are toxin–antitoxin (TA) and restriction–modification (RM) systems (1–4). In TA systems, PSK is caused by different stabilities of cognate antitoxin and toxin moieties (1, 4, 5). At steady state, the toxin is kept inactive by a tightly bound antitoxin. When a plasmid containing a TA module is lost, the less stable antitoxin is degraded, allowing the released toxin molecules to bind/modify their targets and kill plasmid-free cells. RM systems comprise a methyltransferase (MTase) that modifies, by adding a methyl group, a base in the recognition site of DNA and a restriction endonuclease (REase) that recognizes the same site in an unmodified state and cleaves it (6). The most common Type II RM systems typically encode separate REase and MTase polypeptides. When a plasmid carrying a Type II RM system is lost, unmodified DNA sites must ultimately appear in progeny cells due to decay and/or dilution of the MTase. These sites can be attacked by the REase, leading to cell death. The net result is plasmid stabilization. Type II RM systems resemble TA systems, with REase playing the role of a toxin and MTase that of an antitoxin. However, differential stability of RM enzymes, in a few cases when it was studied, was not observed (7, 8). Though this has not been extensively studied, RM systems of other types, Type I and Type III, do not cause PSK (9). The likely reason is that in these systems, multisubunit complexes that contain both the REase and MTase polypeptides are involved in both restriction and methylation reactions.

Though some TA and RM systems clearly stabilize plasmids and many cause PSK, unresolved issues remain. First, in several cases, PSK was studied at conditions of expression of TA systems from inducible promoters, which could have resulted in higher than physiological concentrations of toxic components (10, 11). Second, for some TA systems, PSK was not observed (12). Finally, a “competition hypothesis” states that PSK may mediate exclusion of competing plasmids rather than stabilizing resident plasmids already present in the cell (13–15). In this work, we used the inducible CRISPR-Cas system to rapidly and efficiently eliminate plasmids containing several Type II RM systems from the *E. coli* cells. The EcoRV, Eco29kI, and EcoRI RM systems increased plasmid maintenance and caused very efficient PSK. In contrast, cells that lost plasmids with the Esp1396I system recovered. We show that the unusual behavior of Esp1396I is caused by a short life of the Esp1396I REase activity, which becomes undetectable at or around the time when the second round of chromosomal DNA replication after plasmid loss occurs. Using mathematical modeling, we show that a temporal delay built in Type II RM systems to prevent autoimmunity provides an accessibility window that leads to PSK of cells that lost an RM plasmid even when the lifetimes of RM enzymes are the same. However, the appearance of PSK requires that the restriction endonuclease remains active when unmodified recognition sites start to appear.

## RESULTS

### Type II restriction–modification systems stabilize plasmids

To assess the ability of Type II RM systems to stabilize plasmids in *E. coli* cell populations, pBAD33-g8 vector-based plasmids expressing EcoRV, Eco29kI, EcoRI, and Esp1396I RM system components from their natural promoters were constructed (see Materials and Methods and Table S1). All four plasmids protected *E. coli* KD263 cells from the λ_vir_ phage infection (Fig. S1). Protection levels, calculated as ratios of phage plaques on lawns of plasmid-free KD263 cells to the number of plaques on lawns of cells carrying RM plasmids, decreased from ~7×10^6^ fold for EcoRV to ~5×10^5^ for Eco29kI, ~7×10^4^ for EcoRI, and ~10^3^ for Esp1396I (Fig. S1). The protective activity of different RM systems is apparently unrelated to the number of recognition sites in the phage genome (21 for the EcoRV, four for Eco29kI, five for EcoRI, and 14 for the Esp1396I).

**Fig 1:**
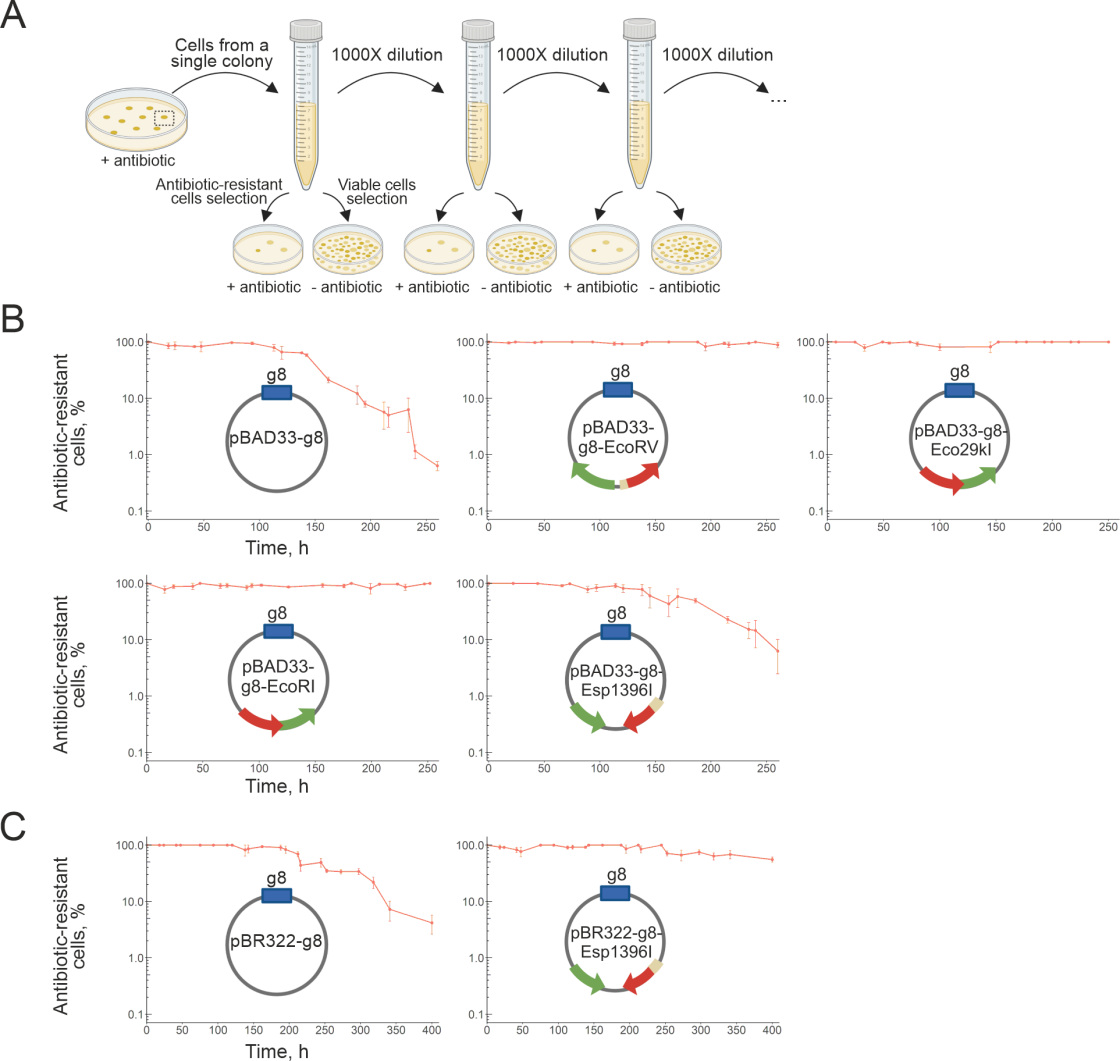
The EcoRV, Eco29kI, EcoRI, and Esp1396I RM systems stabilize plasmids in the absence of antibiotic selection. (**A**) Plasmid stability assay workflow. A single colony of *E. coli* KD263 cells carrying a plasmid under study is grown in LB broth without antibiotics. Cultures are transferred to fresh medium (1:1000 dilution) twice daily. Aliquots are taken before every transfer and plated on LB agar with or without antibiotics to estimate the percentage of antibiotic-resistant cells. (**B**) Percentage of antibiotic-resistant cells carrying control pBAD33-g8 vector (top left) and pBAD33-g8 harboring the EcoRV, Eco29kI, EcoRI, and Esp1396I systems. (**C**) Percentage of antibiotic-resistant cells carrying the control pBR322-g8 vector (left) and pBR322-g8 harboring the Esp1396I system. Mean values and standard errors obtained from three independent experiments are presented.

To measure plasmid stability, cells harboring the empty vector control or plasmids with RM systems were cultivated in liquid Luria–Bertani (LB) medium without antibiotics. Aliquots of cultures were withdrawn twice daily and reseeded in fresh medium. At least 18 reseedings (250 hours) were performed. The percentage of antibiotic-resistant cells in the cultures was monitored at the time of every reseed process by plating cells on LB agar plates with and without selective antibiotics (Fig. 1A).

EcoRV, Eco29kI, and EcoRI systems strongly stabilized the pBAD33-g8 vector (Fig. 1B). Cells with these systems did not lose antibiotic resistance throughout the experiment, while in cultures with the control vector plasmid, plasmid-free cells started to appear after 150 hours (Fig. 1B, left). After 250 hours, only 1% of cells remained antibiotic-resistant. These results agree with data obtained earlier for EcoRV and EcoRI, where slightly different assays were used to monitor plasmid stability (3, 16, 17). The pBAD33-g8-Esp1396I plasmid was only partially stabilized, with ~10% of cells remaining antibiotic-resistant after 250 hours of growth in the absence of antibiotics (Fig. 1B).

Decreased stabilization of plasmids by Esp1396I is apparently unrelated to the number of recognition sites in the host genome (2,041 for EcoRV, 656 for Eco29kI, 646 for EcoRI, and 1,657 for Esp1396I). We considered that the lesser stability of pBAD33-g8-Esp1396I is caused by decreased REase activity. Accordingly, we re-cloned the Esp1396I system on pBR322-g8, which has a higher copy number than that of pBAD33-g8 (~30 versus ~10) (18–20). In agreement with earlier data (18), cells carrying pBR322-g8-Esp1396I were better protected from λ_vir_ than cells carrying pBAD33-g8-Esp1396I (3 × 10^4^ versus 1 × 10^3^ fold, Fig. S1). The pBR322-g8 plasmid was more stable than pBAD33-g8, and cells started losing it only after 200 hours of cultivation with reseeds (Fig. 1C). The pBR322-g8-Esp1396I did not show signs of losing even after 400 hours of cultivation, when less than 10% of cells maintained the empty vector. Thus, similarly to other Type II RM systems, Esp1396I stabilizes plasmids, and the effect depends on plasmid copy number and, therefore, the amounts of RM enzymes.

### CRISPR-mediated loss of plasmids containing some Type II RM systems leads to PSK

**Fig 2:**
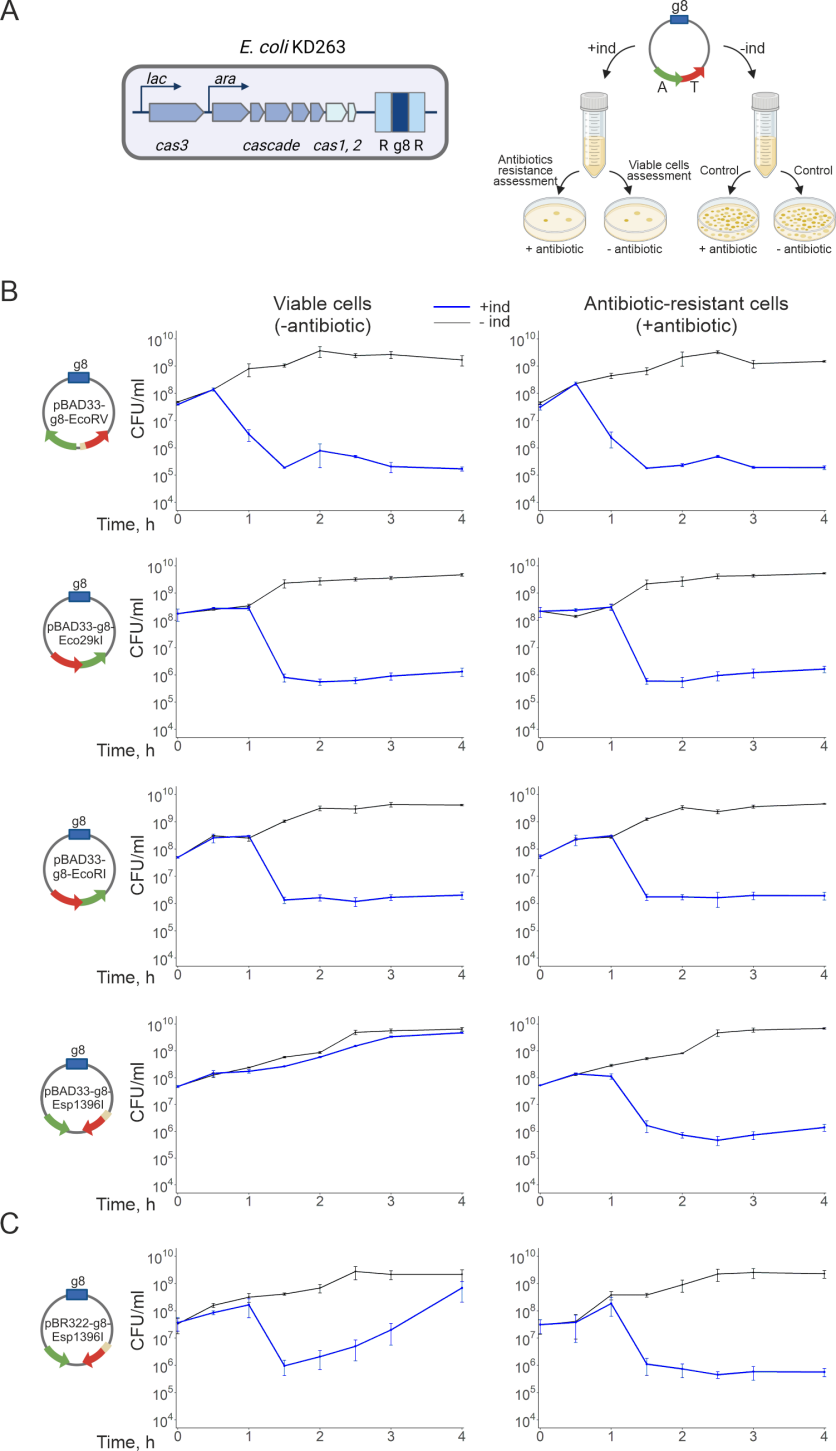
Post-segregational killing upon CRISPR interference induced by loss of Type II RM-bearing plasmids. (**A**) An *E. coli* KD263 cell capable of inducible *cas* gene expression and carrying an engineered CRISPR array with a single g8 spacer (G8) between the repeats (R) is shown on the left. On the right, the PSK assay workflow is schematically presented. KD263 cells containing plasmids with the g8 protospacer and an RM module under study are grown in LB broth without antibiotics. Cultures are induced to activate *cas* genes transcription. Control cultures continue growth without the addition of *cas* gene transcription inducers. Aliquots from both induced and control cultures are withdrawn over the course of 4 hours and plated on LB agar with or without antibiotics to obtain CFU numbers. (**B-C**) Plots demonstrate the dynamics of colony-forming unit (CFU) numbers on plates without (left) and with antibiotics (right) selecting for the presence of pBAD33- (**B**) or pBR322- (**C**) based plasmids carrying indicated RM systems. Blue lines show the number of CFUs in induced cultures, and black lines show uninduced control cultures. Mean values and standard errors obtained from three independent experiments are presented.

The *E. coli* KD263 cells contain a miniature CRISPR array with a single spacer corresponding to a fragment of phage M13 gene 8 (the “g8” spacer) and express *cas* genes upon induction with L-arabinose (Ara) and IPTG (Fig. 2A, see also Ref (21)). The empty vectors and plasmids carrying RM systems contain the g8 protospacer with a functional protospacer adjacent motif (PAM) and are therefore subject to CRISPR interference (22, 23). At our conditions, the yield of plasmids purified from induced cultures is dramatically decreased 30 minutes post-induction (Fig. S2), and 99% of cells lose pBAD33-g8 or pBR322-g8 plasmids 1.5 hours post-induction, as judged by comparing the number of colony-forming units (CFUs) appearing on media with and without the addition of an antibiotic selecting for the presence of the plasmid (Fig. S2).

Expression of *cas* genes in KD263 cultures carrying plasmids containing RM modules was induced, and the total number of viable cells and the number of plasmid-bearing cells was monitored over 4 hours by plating culture aliquots on LB agar with or without selective antibiotics (Fig. 2A, right). As evidenced by the decrease in the number of CFUs formed in the absence of antibiotics, PSK occurred in induced cultures of cells transformed with pBAD33-g8-EcoRV, pBAD33-g8-Eco29kI, and pBAD33-g8-EcoRI (Fig. 2B). Cells from colonies formed on LB plates without antibiotics also formed colonies on antibiotic-containing plates. We conclude that there were no PSK survivors after the loss of EcoRV, Eco29kI, and EcoRI system plasmids. Residual colony-forming cells in induced cultures must have had mutations in their CRISPR-Cas system, in the plasmid protospacer, or failed to respond to inducers. Induction of *cas* gene expression in cultures of cells carrying pBAD33-g8-Esp1396I led to decreased CFUs only on the medium with antibiotics (Fig. 2B, bottom), indicating the absence of PSK.

The loss of the pBR322 vector carrying Esp1396I led to PSK 1.5 hours post-induction (Fig. 2C). Interestingly, while the reduction of viable cell numbers in cultures with the pBAD33-g8-EcoRV, -Eco29kI-, and -EcoRI-plasmids was irreversible (Fig. 2B), cells from cultures with pBR322-g8-Esp1396I lost the ability to form colonies on LB agar plates temporarily, and eventually the numbers of CFUs recovered (Fig. 2C). Cells that accumulated during regrowth were antibiotic-sensitive, again indicating the absence of PSK.

### Cells losing RM system plasmids that cause PSK undergo SOS response

**Fig 3:**
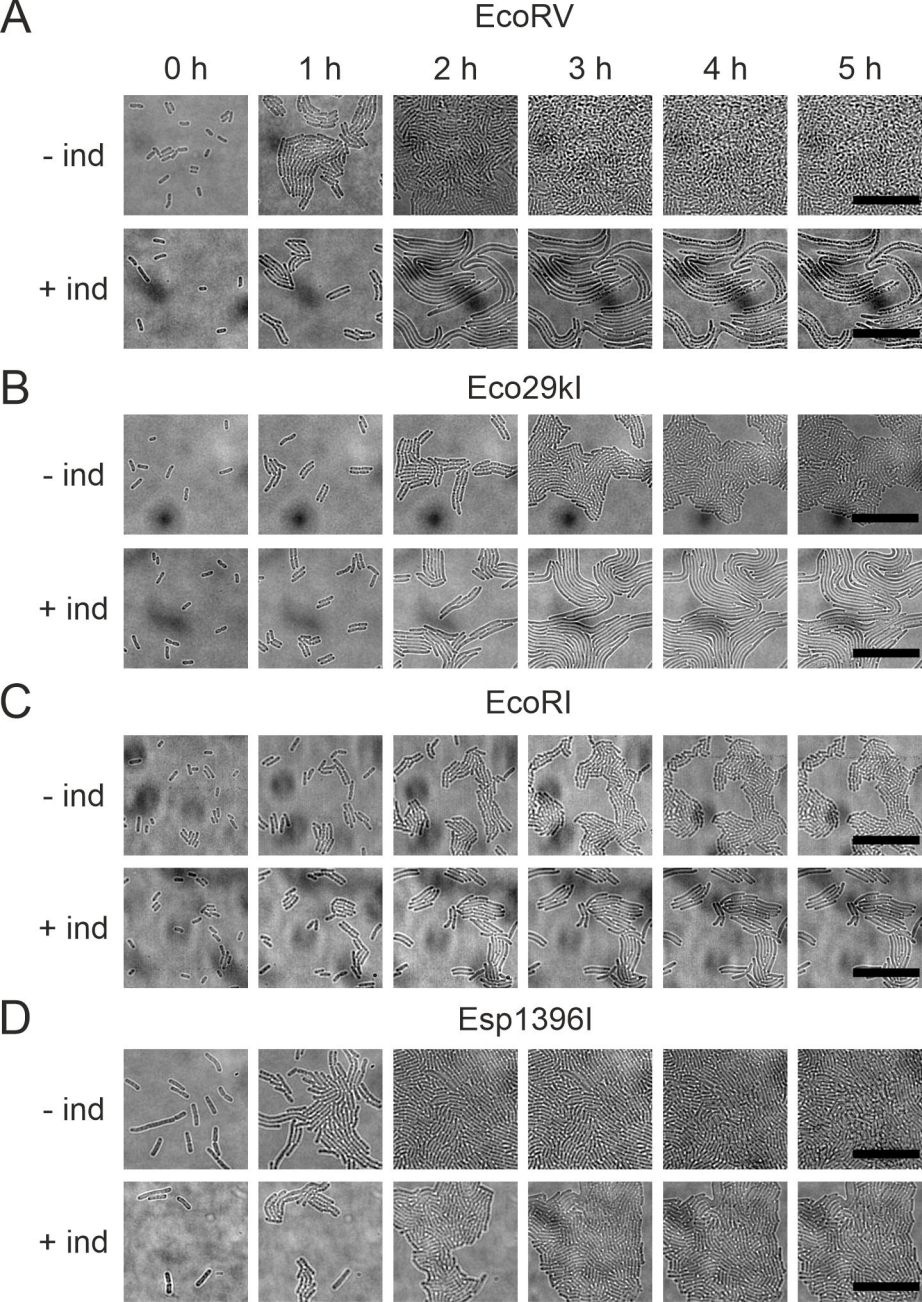
Time-lapse microscopy of growing EcoRV (**A**), Eco29kI (**B**), EcoRI (**C**), and Esp1396I (**D**) carrying cells in the presence (bottom rows of each panel) and in the absence (top rows) of *cas* gene expression inducers. Cells containing plasmids with indicated RM systems were pre-grown for 1 hour in the presence or absence of the *cas* gene inducers, spotted on agarose pads with LB medium with or without inducers and imaged every 15 minutes by time-lapse microscopy in the transmitted light channel. The scale bar corresponds to 20 µm.

We used live microscopy to monitor individual cell morphology and growth upon plasmid loss. Uninduced KD263 cells harboring RM plasmids were spotted on agarose pads with or without inducers and monitored under a microscope in transmitted light. In the absence of inducers, cells actively divided and ultimately formed confluent lawns (Fig. 3A through D, top rows). Upon induction of EcoRV, Eco29kI, and EcoRI plasmid loss, cells divided no more than two times (Fig. S3) and then started forming filaments indicative of DNA damage and SOS response (Fig. 3A through C, bottom rows). In the case of Esp1396I-carrying cells, induced cells continued to divide, and their length did not change compared to control (Fig. 3D, bottom, Fig. S4A). However, the growth of induced Esp1396I carrying cells was slower and, during the time of observation, appeared to be linear rather than exponential (Fig. S4B). Overall, the results agree with data obtained using bulk culture experiments and show that unlike cells that lost the EcoRV, EcoRI, and Eco29kI plasmids, cells that lose the Esp1396I plasmid do not die but undergo temporary growth inhibition.

**Fig 4:**
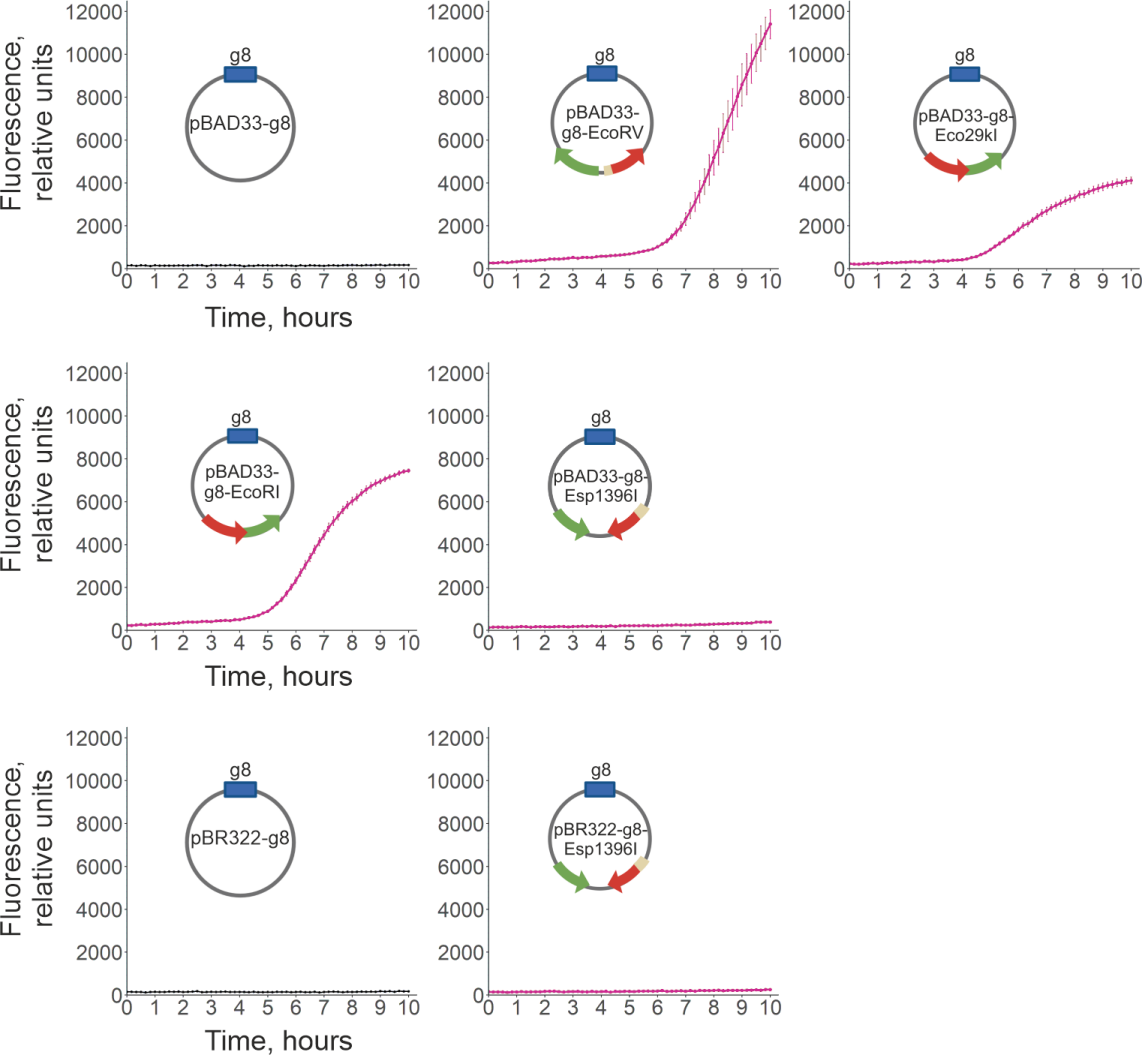
The SOS response is induced in cells that lose plasmids with the EcoRV, Eco29kI, and EcoRI systems. KD263 cells carrying indicated control (pBAD33-g8 or pBR322-g8) or RM plasmids were transformed with a compatible reporter plasmid pDualrep2 expressing RFP upon the SOS response. Plots demonstrate fluorescence intensity change (in relative fluorescence units) in cultures with *cas* gene expression induced at the zero timepoint (see Materials and Methods). Mean values and standard errors obtained from three independent experiments are presented.

To estimate the extent of SOS response in cultures that lost the RM plasmids, we used a compatible reporter plasmid pDualrep2 (24) that contains the *rfp* gene under the control of the SOS-responsive *sulA* promoter (25). In the presence of DNA-damaging agents or replication inhibitors, *E. coli* cells carrying pDualrep2 start to fluoresce (24). We therefore reasoned that in cells undergoing PSK caused by RM, plasmid loss increased fluorescence shall be detected. Indeed, strong induction of fluorescence was observed ~5 hours post-induction of EcoRV, Eco29kI, and EcoRI plasmids carrying cells (Fig. 4). While cultures losing the EcoRV plasmid, which provided the highest protection from phage infection, produced the highest levels of fluorescence, the loss of the Eco29kI plasmid led to a lower fluorescence signal than in the case of the EcoRI plasmid, even though Eco29kI protected cells better than EcoRI. In cells losing the Esp1396I system, fluorescence stayed at the background level, irrespective of the plasmid backbone.

One way to explain the apparently aberrant behavior of the Esp1396I system is to assume that cells are able to repair the DNA damage caused by the Esp1396I restriction endonuclease more efficiently than in the case of systems that cause PSK. To check this idea, we created a *ΔrecA* derivative of KD263. The RecA protein mediates homology recognition and strand exchange during homologous recombination by RecBCD or RecFOR complexes (26, 27), and so neither repair pathways shall be operational in these cells. Cells were transformed with the pBR322-g8-Esp1396I, and the PSK assay was performed. While the mutant cells grew slower than the wild-type, the overall PSK effect (temporal decrease in the number of CFUs followed by recovery) was the same (Fig. S5). Thus, homologous recombination-dependent repair does not contribute to survival of cells that lose a plasmid-borne Esp1396I system.

We sequenced the total genomic DNA of several randomly picked colonies that have lost the Esp1396I plasmid to check whether cells accumulated any mutations. As controls, genomic sequencing of uninduced colonies was performed. No signs of mutations specific to cells that lost the RM plasmid or genomic rearrangements at Esp1396I sites were observed (data not shown).

### The Esp1396I REase activity is short-lived

**Fig 5:**
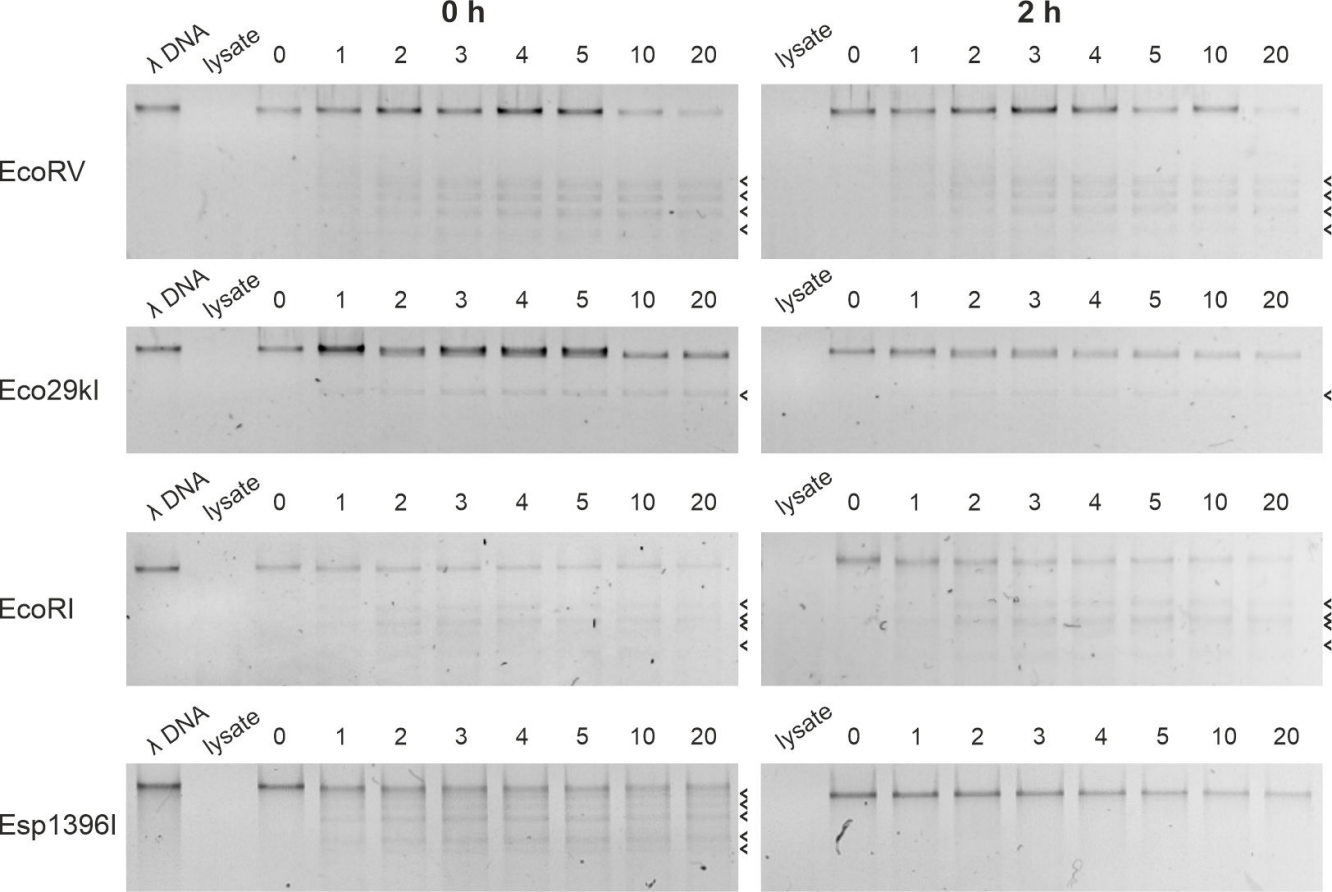
*In vitro* digestion of phage λ DNA in extracts of cells carrying plasmids with indicated RM systems. Extracts prepared from cells carrying plasmids with indicated RM systems were combined with purified λ DNA and incubated for indicated times (in minutes). Digestion reactions were terminated by the addition of EDTA, and samples were analyzed by agarose gel electrophoresis. On panels shown on the left, extracts were prepared from uninduced cells. On panels shown on the right, extracts were prepared from cells 2 hours after the induction of *cas* gene expression. Arrows indicate positions of digestion products specific for each REase. Only fragments of gels containing largest digestion products are shown since bands corresponding to shorter digestion products were not visible. Uncropped gels are shown in Fig. S6.

Experiments presented above demonstrate that three out of four RM systems tested behave in an expected way, and their loss leads to PSK, presumably due to REase activity that is not balanced by MTase activity. The Esp1396I system is distinct, in that cells that lose the Esp1396I plasmid do not undergo SOS response and show no PSK. We hypothesized that the Esp1396I REase activity disappears before unmethylated sites accumulate after RM plasmid loss. Such sites should appear *en masse* after a cell that had lost the RM plasmid divides at least two times since the first division produces hemi-methylated sites that are not subject to restriction. To monitor the activity of REases in cells that lost the RM plasmids, we prepared cell lysates and used them to digest unmodified λ phage DNA. We assumed that MTases, if present, will have no effect on phage DNA digestion due to kinetic differences in MTase and REase activities in cell-free lysates. Phage DNA remained intact for at least 60 minutes of incubation, with cell extracts prepared from KD263 cells without a plasmid or with a plasmid vector (Fig. S6). Though at longer time points a diffused lower-molecular weight smear appeared, no distinct digestion intermediates were observed. In contrast, in reactions containing lysates prepared from uninduced cells with RM system plasmids, phage DNA restriction patterns specific for each REase tested started to appear after the first minute of incubation (Fig. 5, left panels). Extracts of cells carrying RM plasmids collected 2 hours after induction of *cas* gene expression were also prepared and tested. Expected restriction fragments were observed in lysates prepared from cells that originally carried EcoRV, Eco29kI, and EcoRI systems, and the efficiency of cleavage was similar to that in uninduced cell lysates (Fig. 5, right panels). Thus, the REase components of these systems were active even when their synthesis was interrupted by loss of plasmids that carried the systems. In contrast, no digestion was observed in induced lysates of cells that carried the Esp1396I system. More careful monitoring of Esp1396I REase activity decay showed that its activity was lost between 1 and 1.5 hours after induction of *cas* gene expression (Fig. S7). Based on CFU measurements of uninduced cell cultures monitored at the same density as the induced culture, during this time, the cells divide no more than two to three times.

## DISCUSSION

Most Type II RM systems are thought of as addictive modules similar to toxin–antitoxin systems: once a genetic element (e.g., a plasmid) containing an RM system is lost, post-segregational host cell killing should occur due to self-restriction at unmodified recognition sites (2, 3, 15, 28, 29).

Currently, there are two widely used approaches to remove plasmids with addiction modules from cells for PSK studies: heat shock of cells bearing the plasmid with temperature-sensitive replicons (2, 4, 30, 31) or conditional prevention of plasmid replication (3, 32). Recently, a new system to purge specifically designed plasmids by targeting them with an inducible meganuclease has been described (33). In this work, we used a similar concept and harnessed inducible CRISPR-Cas interference to synchronously and rapidly eliminate plasmids with RM modules from *E. coli* cells. We used this approach to study the consequences of loss of plasmids carrying four Type II RM systems: EcoRV, EcoRI, Eco29kI, and Esp1396I. These systems were discovered on low-copy number natural plasmids of *E. coli* (EcoRV, EcoRI, and Eco29kI) or *Enterobacter* sp. (Esp1396I) (34–37). EcoRV and EcoRI were previously shown to stabilize plasmids and cause PSK (3, 7, 16, 28, 38). We observed strong stabilization of plasmids by EcoRV, EcoRI, and Eco29kI. In contrast, Esp1396I stabilized plasmids weakly or not at all, depending on plasmid copy number. The result could not be due to the lower number of Esp1396I recognition sites in the *E. coli* genome, which exceeds those for Eco29kI and EcoRI more than 2.5-fold.

Stronger stabilization of higher copy number Esp1396I plasmids is consistent with data obtained with EcoVIII, which stabilized a high-copy number plasmid better than a low-copy one (39). Even when no plasmid stabilization was observed, Esp1396I efficiently restricted phage λ_vir_ growth. Similar results were previously obtained with the Bsp6I RM system, which protected cells from phage λ infection, but did not appreciably stabilize a plasmid that carried it during long-term cultivation (3).

The loss of EcoRV, EcoRI, and Eco29kI plasmids by targeting them with CRISPR-Cas led to PSK: cells became filamentous, SOS response was induced, and a strong loss of viability was observed. In contrast, the loss of lower-copy number Esp1396I plasmids resulted in no PSK, while the loss of a higher-copy number plasmid caused a temporal loss of CFUs followed by full recovery. Live microscopy of cells losing either Esp1396I plasmid did not show increase in individual cell lengths, and no SOS induction was detected.

After cleavage, the EcoRV REase generates blunt DNA ends, while EcoRI and Eco29kI generate sticky complementary ends with 5’- or 3’-overhangs, respectively. The Esp1396I REase recognizes an interrupted palindromic sequence and generates sticky ends with 5’-overhangs, which are noncomplementary for different sites. We considered that the lack of PSK in cells losing the Esp1396I plasmid could be due to a more robust repair of more recombinogenic cleaved Esp1396I sites by the repair machinery of the cell. This could have been possible since cells losing plasmids carrying the EcoRI or PaeR7 systems demonstrated more severe growth inhibition and filamentous phenotype on the background of *recBCD* mutations (2, 7, 28). Yet growth inhibition of cells losing the high-copy number Esp1396I plasmid remained reversible on the background of a *recA* deletion. Thus, it appears that cells that lose Esp1396I plasmids survive because there is no self-restriction.

In the case of toxin–antitoxin systems, it is generally accepted that PSK, when it is observed, is explained by a shorter lifetime of the antitoxin, which allows accumulation of free toxins after toxin–antitoxin plasmid loss with subsequent death of the cell. A similar mechanism may be operational with RM systems since decrease in intracellular concentrations of protecting methyltransferases caused by both dilution during cell growth/division and by proteolytic degradation should ultimately expose genomic targets of the newly replicated chromosome for the REase attack. However, to the best of our knowledge, no data showing lesser stability of MTase for Type II RM systems are available. In fact, in a few cases where the stabilities of cognate MTases and REases were studied, they were found to be similar (7, 8).

A principal difference in the ways TA and RM systems operate may explain these observations. A proper function of an RM system requires that hemi-methylated sites produced upon replication of fully methylated DNA are completely methylated by an MTase before a new round of replication begins. This lag in accessibility to REase-catalyzed cleavage allows a cell to avoid self-restriction while simultaneously maintaining the ability to reliably eliminate non-methylated incoming DNA during exponential growth. Shortly after RM plasmid loss, the activity of MTase is close to its initial steady-state level, and the probability of non-methylation of hemi-methylated sites that appear during the subsequent round of bacterial chromosome replication is minimal. At later times, the activities of both enzymes decay, and at some point, the REase activity will reach such a low level that none of unmethylated sites (which appear due to decay in MTase activity) are cut. Thus, the probability of site cleavage (and, therefore, PSK induction) should reach a maximum at some point after the RM plasmid loss, even when the REase and MTase activity decay at the same rate.

**Fig 6:**
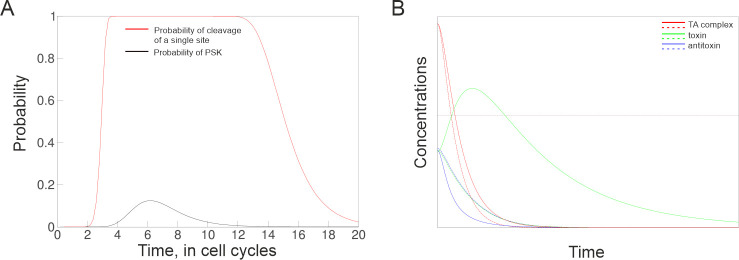
Modeling PSK mediated by RM (**A**) and TA (**B**) system plasmid loss. Plasmid loss occurs at a zero timepoint. Panel (**A**) shows the calculated probability of cleavage of a single site (black line) and of PSK (restriction of at least one of 1,000 sites in bacterial chromosome, red line) vs time when the first hemi-methylated site appeared. The parameters of the model are presented in the “Deriving a model of RM-based PSK” subsection of Materials and Methods. Results of modeling for identical decay rates of REase and MTase activities are shown. Panel (**B**) shows changes in the toxin concentration upon the loss of a plasmid encoding a classical TA system. Solid lines show the concentrations of toxin (green), antitoxin (blue), and toxin–antitoxin complex (red) in the case when the decay of antitoxin is four times faster than that of the toxin. Dashed lines show changes of same components when toxin and antitoxin have identical decay rates. The prominent spike in the concentration of free toxin caused by faster decay of antitoxin is absent when decay rates are identical.

To quantitatively confirm these intuitive considerations, mathematical modeling was performed. We evaluated the probability that a recognition site is not affected by an enzyme whose concentration decays exponentially due to dilution and/or degradation (see Eq. (4) in Methods). Next, we derived an expression for probability of a sequence of events that consists of non-methylation of a hemi-methylated site, followed by subsequent cleavage, rather than methylation of a non-methylated site that is produced from a hemi-methylated site after a round of replication (equation. (12)). Finally, we followed this probability as a function of time (equation. (13)), expressed in cell cycles after elimination of RM plasmids. As can be seen from Fig. 6A, for identical decay rates of REase and MTase activities, the probability of restricting a single target site indeed reaches a maximum after a certain time after plasmid loss and then declines. Importantly, even a low per-site probability of cleavage ensures PSK, given the large (∼10^3^) number of recognition sites in a bacterial genome. The observed behavior is in marked contrast to modeling obtained for a case of a classical TA system, where PSK is assumed to occur without a lag of accessibility, but rather be an immediate consequence of accumulation of a threshold amount of free toxin liberated from a toxin–antitoxin complex. As can be seen from Fig. 6B, if toxin and antitoxin have identical half-lives, a loss of a TA system plasmid cannot lead to PSK because cells will simply experience a gradual decrease of the initial free toxin level that exists in the presence of the TA system plasmid. In contrast, faster decay of the antitoxin results in a prominent spike in the concentration of the free toxin that could be sufficient to kill and/or significantly reduce the growth of cells that have lost a plasmid. Obviously, the magnitude of free toxin spike will increase with higher steady-state concentrations of the toxin–antitoxin complex. Therefore, PSK in the case of TA systems should depend on the copy number of plasmids that encode them, as is indeed observed (40).

If the time that the activity of REase sufficient to produce enough cuts in unmodified sites is shorter than the accessibility window, then no PSK is expected, irrespective of MTase activity levels at any given time. We propose that this factor explains the unusual behavior of Esp1396I, whose REase effective activity persists for a shorter period of time than required for two rounds of DNA replication at our conditions. If the REase activity disappears by the beginning of the second round of replication after plasmid loss, no PSK is expected, irrespective of MTase activity levels. We note that the relevant effective REase activity is a cumulative function of REase peptide stability and the specific activity of the enzyme toward its recognition sites. Importantly, the shorter effective REase activity lifetime should not affect the efficiency of protection from phage infection of cells carrying an RM plasmid as long as a sufficient steady-state activity level (which again is a function of REase protein concentration and its specific activity) is maintained.

The accessibility window that determines whether PSK is observed upon RM plasmid loss should depend on host DNA replication rate. For example, we predict that increasing the growth rate may promote PSK for Esp1396I. The accessibility window may also depend on the host (either because of different replication rates or different RM enzyme half-lives). Indeed, the Bsp6I RM system was shown to stabilize plasmids in its natural host *B. subtilis,* but not in *E. coli* (3). Be that as it may, our analysis shows that the difference in TA and RM system action, the former acting immediately after toxin release, while the other experiencing a built-in delay in self-restriction, ensures robust RM-mediated PSK at similar REase and MTase activity decay rates as long as the life of REase activity persists is longer that the time needed to replicate a significant number of recognition sites twice.

## MATERIALS AND METHODS

### Bacterial strains, plasmids, and oligonucleotides

*E. coli* KD263 (K-12 F+, *lacUV5-cas3 araB*p8-*cse1*, CRISPR I: repeat-spacer g8-repeat, CRISPR II deleted) (21) was used throughout in plasmid loss experiments. *E. coli* BL21 (AI) (B F- *ompT hsdS*_B_(r_B_^-^ m_B_^-^) *gal dcm araB::T7RNAP-tetA*) was used for cloning. *E. coli* KD263 with a deletion of the *recA* gene was used in PSK experiments with the pBR322-g8-Esp1396I plasmid. This strain was constructed from parental KD263 by transduction with the phage P1 lysate prepared on a *ΔrecA* strain from the Keio collection (41, 42). The parental deletion is marked with a kanamycin resistance gene, and transductants were selected on the medium containing 100 µg/mL kanamycin and confirmed by PCR and Sanger sequencing of amplicons.

All plasmids and synthetic oligonucleotides used are listed in Tables S1 and S2. Plasmids were constructed by the Gibson assembly method using NEBuilder HiFi DNA Assembly Master Mix (New England Biolabs). Details of construction of each plasmid are provided in Table S1. The pDualrep2 reporter plasmid (24) was kindly provided by Dr. Ilya Osterman.

### Bacterial growth

*E. coli* cells were grown in standard LB medium supplemented with antibiotics and inducers (if necessary) or on LB agar plates containing 1.5% agar with antibiotics (100 µg/mL ampicillin (Amp), 34 µg/mL chloramphenicol (Cm), 50 µg/mL kanamycin (Kan)) and 1% D-glucose (Glc) if necessary. L-arabinose (Ara) and isopropyl-β-d-1-thiogalactopyranoside (IPTG) were used in 1 mM concentrations each. To prevent leaky expression from the *araBAD* promoter, overnight bacterial cultures were grown in the presence of 0.2% Glc. After transformation, cells were recovered in the SOC medium (Super Optimal broth with catabolite repression).

### CRISPR interference assay

Overnight cultures of KD263 cells containing plasmids with or without the g8 protospacer were diluted 50 times and grown until OD_600_ = 0.3 without antibiotics. At this point, half of the cells were induced with Ara and IPTG. Another half of the culture remained uninduced. Cultivation was continued, and aliquots were withdrawn immediately prior to induction and at 30-minute intervals after the induction for 5 hours. The colony-forming unit (CFU) numbers were obtained by spotting 5-µL drops of serial tenfold dilutions (in 10 mM MgSO_4_) of withdrawn aliquots on the surface of LB agar plates with or without antibiotics. The plates were supplemented with Glc to stop *cas* gene transcription. All experiments were performed at least in three biological replicates. Plasmid loss was observed by purifying plasmids from 2-mL culture aliquots using the GeneJET Plasmid Miniprep Kit (Thermo Scientific) followed by electrophoresis in 0.8% agarose gels.

### The PSK assay

Overnight cultures of KD263 harboring plasmids of interest were diluted 50 times and grown until OD_600_ = 0.3 without antibiotics. Half of the culture was induced with Ara and IPTG, and another remained uninduced and served as a control. Both cultures were grown for 4 hours with aliquots withdrawn every half hour, and CFU numbers were determined as described above. All experiments were performed at least in three biological replicates.

### Plasmid stability assay

A single colony of KD263 cells carrying a plasmid of interest was grown overnight in 5 mL LB without antibiotics. Cultures were diluted 1:1000 in fresh LB, and the cultivation was continued. The dilution step was repeated twice daily. Twenty-microliter aliquots were taken before every transfer, diluted in 10 mM MgSO_4_, and 5-µL drops of serial tenfold dilutions were spotted on LB agar with or without antibiotics. Plasmid stability was estimated as the percentage of antibiotic-resistant cells: $\frac{CFU\;on\;LB\;with\;antibiotic}{CFU\;on\;LB\;without\;antibiotic}\times 100\%$. All experiments were performed at least in three biological replicates.

### Phage plaquing assay

Bacteriophage λ_vir_ lysates were prepared as described elsewhere (43). Overnight cultures of KD263 cells without plasmids (control) or harboring plasmids with the EcoRV, Eco29kI, EcoRI, or Esp1396I RM systems were diluted 100 times and grown until OD600 = 0.4. Petri dishes containing 10 mL of 1.5% bottom LB agar were cast with 4 mL 0.4% top LB agar supplemented with appropriate antibiotics, 10 mM MgSO_4_, 0.2% maltose, and 150 µL of cell cultures. Five-microliter drops of serial tenfold λ_vir_ phage lysate dilutions were spotted on the surface of top agar, allowed to dry, and plates were incubated at 37°C overnight. The phage titer was determined by counting distinct plaques at the highest phage lysate dilution. Protection levels were calculated by dividing the determined titer of the phage lysate on the lawn of plasmid-free KD263 cells by a titer determined on lawns of cells carrying plasmids of interest. All experiments were performed at least in three biological replicates.

### Microscopy

Cells were observed using an inverted light microscope Nikon Eclipse Ti-E. The time-lapse was recorded with the Andor ZYLA 4.2MP Plus camera.

To make microscopy slides, the double-sided tape was attached to the microscope slide (25 × 75 mm) to form the frame. A volume of 500 µL of 1.5% agarose diluted in LB was placed inside the frame and sealed by pressing with the second microscope slide. If necessary, inducers (Ara and IPTG) were added to agarose in concentrations of 1 mM each. After agarose solidification, the upper slide was removed.

Overnight cultures of KD263 cells transformed with plasmids of interests were diluted 50 times and grown until OD_600_ = 0.3 without antibiotics. Half of the culture was induced, and another remained uninduced. The cultivation was continued for 1 hour, and 1-µL culture aliquots were spotted onto agarose pads with or without inducers. When the sample was fully absorbed, the chamber was covered with a 24 × 40 mm coverslip and sealed with petrolatum, lanolin, and paraffine mixture to prevent agarose drying. Filming was carried out every 15 minutes for at least 5 hours in a transmitted light (TL) channel. Samples were kept at the microscope observation stage at 37˚C during the entire observation time.

Image analysis for Fig. S3 was performed manually by calculating the total number of cells in the field of view and percentage of cells dividing once or twice before elongation. Number of cells used for calculations was >50 for each system.

### SOS response detection

Overnight cultures of KD263 cells containing RM-system plasmids or control vector and the pDualrep2 plasmid (24) were diluted 50 times and grown until OD_600_ = 0.3 without antibiotics. Two hundred microliters of each culture (in triplicate) was placed in a 96-well plate and induced with Ara and IPTG. The plate was placed into EnSpire Multimode Plate Reader (Perkin Elmer), and cell growth (OD_600_) at 37˚C and RFP fluorescence (553/574 nm) were monitored for 10 hours. The autofluorescence background determined as fluorescence of KD263 cells without plasmids was subtracted.

### Restriction of λ phage DNA in cell-free extracts

Overnight cultures of control (without plasmid) KD263 cells or cells containing RM plasmids were diluted 50 times and grown in 10 mL of LB until OD_600_ = 0.3 without antibiotics. A 5-mL aliquot (a zero timepoint) was withdrawn, and remaining cells were induced with Ara and IPTG. Cultivation was continued for 2 hours, cultures were diluted to OD_600_ = 0.3, and 5-mL aliquots of each culture were withdrawn (2 hours timepoint). Cells were collected by centrifugation, and the pellet was resuspended in 1 mL PBS (Sigma-Aldrich). Cell lysates were obtained by sonication (20% amplitude, 1 minute of pulse time on Qsonica sonicator with 2 mm sonotrode). Five microliters of the lysate was added to phage λ DNA reaction mix (81 µL of H_2_O, 10 µL of 10X FastDigest buffer (Thermo Scientific), and 4 µL of λ DNA (50 ng/µl, *dam-*, *dcm-*; SybEnzyme)). Reactions were allowed to proceed at 37˚C for 20 minutes. Ten-microliter aliquots were withdrawn every minute during the first 5 minutes of incubation and at 10- and 20-minute timepoints. Reactions were terminated by the addition of 1 µL of 0.1M EDTA. Reaction products were resolved by electrophoresis in 0.7% agarose gels.

### Deriving a model of RM-based PSK

Upon targeting by CRISPR interference, g8 protospacer plasmids carrying RM systems are very quickly cleared from the cell. Starting from that moment, the concentrations of restriction endonuclease (REase, [RE]) and methyltransferase (MTase, [MT]) decay due to degradation and dilution during cell division. We assume both these processes to be continuous in time with first-order kinetics, such that expressions for enzyme concentrations are


(1) $$\begin{array}{cc} \frac{d\left[RE\right]}{dt} & =-\rho \left[RE\right],\\ \frac{d\left[MT\right]}{dt} & =-\mu \left[MT\right] \end{array}$$

which results in exponential decay:


(2) $$\begin{array}{cc} \left[RE\right]\left(t\right) & ={\left[RE\right]}_{0}\exp\left(-\rho t\right)\;\\ \left[MT\right]\left(t\right) & ={\left[MT\right]}_{0}exp\left(-\mu t\right).\; \end{array}$$

Here, ρ, µ, and $[RE{]}_{0}$, $[MT{]}_{0}$ are the decay constants and the initial concentrations of corresponding enzymes.

Consider a target site in the bacterial genome that can be methylated or cleaved by the REase. First assume that only one enzyme type (e.g., MTase) is present in the cell. The decay of probability $P_{NMT}(t)$ that the target site has not been affected by the enzyme (not methylated) by time t is given by the following kinetic equation:


(3) $$\begin{array}{cc} \frac{dP_{NMT}\left(t\right)}{dt} & =-k_{MT}\left[MT\right]\left(t\right)P_{NMT}\left(t\right).\; \end{array}$$

It is proportional to the probability to survive by this time (a necessary condition) and the per molecule rate of decay, which is proportional to the enzyme concentration $[MT{]}_{t}$ and is characterized by the methylation rate constant $k_{MT}$.

Taking into account the exponential form of ${\left[MT\right]}_{t}$ , we obtain


(4) $$\begin{array}{c} P_{NMT}\left(t\right)=\exp\left[-\frac{{\left[MT\right]}_{0}k_{MT}}{\mu }\left(1-e^{-\mu t}\right)\right].\; \end{array}$$

Evidently, a similar result would have held in the situation when only the REase enzymes were present if not for the fact that REase is only active as a homodimer. In this study, we assume that dimerization binding is strong, so the concentration of a dimer is half of the total concentration of REase. Absorbing the factor 1/2 into the definition of $k_{RE}$, we obtain


(5) PNREt=exp⁡-[RE]0kREρ1-e-ρt.

It is convenient to denote the product of initial enzyme concentration and its corresponding reaction constant by a single symbol,


(6) $$\begin{array}{cc} m & \equiv [MT{]}_{0}k_{MT},\\ r & \equiv [RE{]}_{0}k_{RE.} \end{array}$$

Constants mandr have the dimension of inverse time ($hour^{-1}$) and describe the rates of methylation and cleavage of a target site for initial steady-state concentrations of corresponding enzymes in the presence of an RM plasmid.

An interesting observation is that because of the decay of enzyme concentrations, the long-time probability for a site to remain unaffected by an enzyme is finite rather than 0,


(7) $$\begin{array}{c} P_{NMT}\left(t⟶\infty \right)=\exp\left[-\frac{m}{\mu }\right] \end{array}.$$

When both REase and MTase enzymes are present in the cell, the probability that a site remains unaffected by either enzyme up to time t is defined by


(8) $$\begin{array}{cc} \frac{dP_{NMTRE}\left(t\right)}{dt} & =-\left\{k_{MT}\left[MT\right]\left(t\right)+k_{RE}\left[RE\right]\left(t\right)\right\}P_{NMTRE}\left(t\right) \end{array}$$

with the solution


(9) $$\begin{array}{c} P_{NMTRE}\left(t\right)=\exp\left[-\frac{r}{\rho }\left(1-e^{-\rho t}\right)-\frac{m}{\mu }\left(1-e^{-\mu t}\right)\right]. \end{array}$$

Now, we evaluate the probability of a scenario when a hemi-methylated site created upon a DNA replication event remains hemi-methylated during the whole replication cycle due to insufficient MTase activity caused by its decay. The next DNA replication event creates non-methylated recognition sites that can be targeted by REase. In the event when REase binds to such a site first ahead of MTase, cleavage occurs. We choose to parametrize this probability by the time t when the non-methylated site becomes accessible to REase (counted from the moment of elimination of plasmids). The right-hand side of the differential equation for this probability,


(11) $$\begin{array}{c} \frac{dP_{RE}}{d\theta }=\exp\left[-\frac{re^{-\rho t}}{\rho }\left(1-e^{-\rho \theta }\right)-\frac{me^{-\mu t}}{\mu }\left(1-e^{-\mu \theta }\right)\right]re^{-\rho \left(t+\theta \right)}, \end{array}$$

consists of two terms: the first term with the exponential function reflects the probability that from time t to time t+θ , the site has neither been cleaved nor methylated. It is similar to (9), but considers the decay of concentrations of REase and MTase, which become $re^{-\rho t}$ and $me^{-\mu t}$ at the beginning of the interval. The second term that follows the square brackets describes the rate of cleavage at the moment t+θ.

The solution of this equation, given by the integral of its right-hand side with respect to time θ, takes a simple analytic form when the decay rates of both enzymes are identical, μ=ρ:


(12) $$\begin{array}{c} P_{RE}\left(t,\theta \right)=\frac{r}{r+m}\left\{1-\exp\left[-\frac{\left(r+m\right)e^{-\rho t}}{\rho }\left(1-e^{-\rho \theta }\right)\right]\right\} \end{array}$$

Note that a common reason for identity of decay rates of both enzymes could be that their concentrations are predominantly affected by dilution. In this case, μ=ρ=ln⁡2/τ, where τ is the duration of the cell cycle.

To determine the probability $P_{1}$ of the sequence of events resulting in cleavage of a single site, we need to multiply the probability $P_{RE}$ (12) of cleavage of the non-methylated site by the probability $P_{NMT}$ (4) that the ancestral site remained hemi-methylated (unaffected by MTase) during the preceding DNA replication cycle.


(13) $$\begin{array}{cc} & P_{1}\left(t,\theta \right)=\exp\left[-\frac{me^{-\rho t}}{\rho }\left(1-e^{-\rho \tau }\right)\right]\times \\ \frac{r}{r+m} & \left\{1-\exp\left[-\frac{\left(r+m\right)e^{-\rho \left(t+\tau \right)}}{\rho }\left(1-e^{-\rho \theta }\right)\right]\right\}. \end{array}$$

There are three time-like arguments in (13): time t, the beginning of the DNA replication cycle that resulted in the site remaining hemi-methylated; τ, the duration of the replication cycle; and θ, the duration of the time interval in the following DNA replication cycle when cleavage occurs. The plot of probability $P_{1}$ of cleavage of a single target site (13), together with the probability of cutting any of $N=10^{3}$ sites in the bacterial genome, $P_{PSK}=1-(1-P_{1}{)}^{N}$, is shown in Fig. 6A. The parameters used in the plot are μ=ρ=ln⁡2/τ, r=m=100ρ, and θ=τ. Varying r/m in a reasonable range (see below) does affect $P_{1}$ but still results in a PSK “overkill”, $P_{PSK}=1$. A change in r/ρ (while keeping r/m constant) does not change the maximal probability, as we prove below.

It is interesting to find the maximum of (13) with respect to time t (or the number of DNA replication cycles when this sequence of events is most probable). To do so, we differentiate (13) with respect to t (or, equivalently, $e^{-\rho t}$) and look for $t^{*}$ that makes the derivative equal to 0.

The resulting algebra is simple but bulky and is not presented here. The solution for the maximum probability is


(14) $$\begin{array}{c} P_{1}^{*}\left(\theta \right)=\beta {\left(1+\beta \right)}^{-1/\beta -1}\frac{r}{r+m},\\ where\;\;\beta \equiv \frac{r+m}{m}\frac{1-e^{-\rho \theta }}{e^{\rho \tau }-1}. \end{array}$$

It turns out that under our assumptions, the initial activities of REase and MTase appear in (14) only as a ratio. Primarily, it means that the effect of plasmid copy number on the probability of PSK is "indirect" and limited to the fairly small differences in REase to MTase activity ratios r/m between various plasmid systems. The range of r/m for Esp1396I-plasmids used in this study can be found, for example, in Ref. (18) (r/m =0.2–1.25). We also assume that the decay of concentration of RM enzymes is only due to dilution, exp⁡(ρτ)=2, and the time interval when cleavage of a non-methylated site could take place is assumed to be confined between two limits, the cell cycle duration, θ=τ (and therefore exp⁡(-ρθ)=1/2), and very long time, θ→∞. Under those assumptions, $P_{1}^{*}$ is confined between 0.05 and 0.2. Taking into account the large number of RM sites in the bacterial genome, the shown values of single site cleavage probability almost certainly guarantee PSK even with ongoing DNA repair. Qualitatively, this explains our observations with EcoRV, Eco29kI, and EcoRI plasmids.

### A model of TA-based PSK

We present a schematic description of PSK after loss of plasmids that contain toxin–antitoxin (TA) modules. For brevity, we denote plasmids as P, toxin as T, antitoxin as A, and toxin–antitoxin complex as C. We assume that plasmids containing the TA system are eliminated at a certain moment $t_{0}$. The TA molecules are produced only in the presence of the plasmid and are constantly degraded both in their free and bound state into the complex forms. Specifically, we consider the following processes with corresponding rates

Decay of plasmids, $P\overset{\delta_{P}}{\to }0$,

Production of toxin, $P\overset{\beta_{T}}{\to }P+T$,

Production of antitoxin, $P\overset{\beta_{A}}{\to }P+A$.

Binding of toxin and antitoxin into the complex, $T+A\overset{k_{A}}{\to }C$.

Dissociation of the complex, $C\overset{k_{D}}{\to }A+T$,

Degradation of free toxin, $T\overset{\delta_{T}}{\to }0$.

Degradation of free antitoxin, $A\overset{\delta_{A}}{\to }0$,

Degradation of bound toxin, $C\overset{\delta_{T}}{\to }A$.

Degradation of bound antitoxin, $C\overset{\delta_{A}}{\to }T$

The changes in concentrations that result from these processes are described by a system of kinetic equations,


(15) $$\begin{array}{c} \frac{dP}{dt}=-\delta_{P}P,t>t_{0}\\ \frac{dT}{dt}=\beta_{T}P-\delta_{T}T+k_{D}C-k_{A}AT+\delta_{A}C\\ \frac{dA}{dt}=\beta_{A}P-\delta_{A}A+k_{D}C-k_{A}AT+\delta_{T}C,\\ \frac{dC}{dt}=k_{A}AT-k_{D}C-\left(\delta_{T}+\delta_{A}\right)C. \end{array}$$

These equations indicate that for PSK to take place, the decay rate of antitoxin has to be significantly larger than that of the toxin $\delta_{A}\gg \delta_{T}$. To avoid toxicity at the beginning of the production of toxin and antitoxin, the production rate of antitoxin should be larger as well, i.e., $\beta_{A}\gg \delta_{T}$. Finally, the binding of toxin to antitoxin should be sufficiently strong so that the corresponding dissociation constant $K_{D}\equiv k_{D}/k_{A}\ll C.$ It is also assumed that formation and dissociation of the toxin–antitoxin complex are fast so that both $k_{A}$ and $k_{D}$ are sufficiently large. The following numerical values of rate constants are assumed:

$\delta_{P}=1$ (this sets the time scale),$\beta_{A}=0.2,\;\delta_{A}=0.2,\;\beta_{T}=0.05,\;\delta_{T}=0.05,\;k_{D}=10,\;k_{A}=100\text{. }$

The equation. (15) are integrated numerically for initial plasmid concentrations, $P_{0}=1.$

It follows from the plots presented by solid lines in Fig. 6B that the spike in the concentration of toxin caused by faster elimination of antitoxin is high enough to exceed the lethal threshold, assumed here to be 0.4. However, when the decay rates of toxin and antitoxin are the same, $\beta_{T}=\beta_{A}=0.2$, there is no spike in the concentration of toxin, and PSK does not occur (Fig. 6B, dashed lines).

## Data Availability

The data underlying this article are available in the article and in its online supplemental material. Bacterial strains and plasmids used in this study can be provided upon request.

## SUPPLEMENTAL MATERIAL

Supplemental figures and tables mbio.01408-24-s0001.docx Tables S1 and S2; Fig. S1 to S7.

ASM does not own the copyrights to Supplemental Material that may be linked to, or accessed through, an article. The authors have granted ASM a non-exclusive, world-wide license to publish the Supplemental Material files. Please contact the corresponding author directly for reuse.
