# Supplementary material for: Dependence of post-segregational killing mediated by Type II restriction–modification systems on the lifetime of restriction endonuclease effective activity
Source: mBio. 2024 Jul 9;15(8):e01408-24. doi: 10.1128/mbio.01408-24 (PMC11324026; doi:10.1128/mbio.01408-24)
Supplement: Supplemental figures and tables — Tables S1 and S2; Fig. S1 to S7. [file mbio.01408-24-s0001.docx]

**Supplementary materials**

**Table S1. List of plasmids used in this work.**

| **Plasmid** | **Description** | **Details of construction / Source** | **Map** |
| --- | --- | --- | --- |
| pBAD33 | p15A ori; Cm^R^ | Lab collection, (1) | https://benchling.com/s/seq-rMQyBFsA6mPrAbkC5O9p?m=slm-nc0plua9KBvCkq0m62s4 |
| pBAD33-g8 | pBAD33 with g8 protospacer with PAM; *araC* gene disrupted | Lab collection, a pBAD33 derivative harboring a g8 protospacer cloned via *HindIII* and *AflII* restriction sites. The *araC* gene of pBAD33 backbone was disrupted during cloning. | https://benchling.com/s/seq-abFXM2u3PmyI6LwfkBSy?m=slm-IwqgTDHYlW01Bo5QxsVt |
| pBAD33-g8-EcoRV | pBAD33-g8 with the EcoRV system; *araC* gene disrupted | A linearized pBAD33-g8 vector was prepared by inverse PCR (primers pBAD_EcoRV_GA_F, pBAD_EcoRV_GA_R**)** and assembled with a PCR product containing the entire EcoRV RM system amplified from the pEF42 plasmid (2) (primers EcoRV_frag_F, EcoRV_frag_R). | https://benchling.com/s/seq-gKAGAdCYOFnZA20LArIO?m=slm-vcf7daFRnxEbdVbwFGaz |
| pBAD33-g8-Eco29kI | pBAD33-g8 with the Eco29kI system; *araC* gene disrupted | The pBAD33-g8 vector linearized as described for pBAD33-g8-EcoRV construction (above) was assembled with a PCR product containing the entire Eco29kI RM system amplified from the pECO29 plasmid (3) (primers Eco29_F, Eco29_R). | https://benchling.com/s/seq-pMy1MQrJ04N3H36IZ3Oe?m=slm-UeZ3I7myekOoRnPyALTn |
| pBAD33-g8-EcoRI | pBAD33-g8 with the EcoRI system; *araC* gene disrupted | The pBAD33-g8 vector linearized as described for pBAD33-g8-EcoRV construction (above) was assembled with a PCR product containing the entire EcoRI RM system amplified from the pR1 plasmid (a gift from Dr. M. Zakharova, Institute of Biochemistry and Physiology of Microorganisms, Russian Academy of Sciences, Pushchino, Russia) (primers EcoRI_F, EcoRI_R). | https://benchling.com/s/seq-xb7H3xCQd4b5StwCYmaK?m=slm-VAlG4Q2G7KrOYub4sKl1 |
| pBAD33-g8-Esp1396I | pBAD33-g8 with the Esp1396I system; *araC* gene disrupted | The pBAD33-g8 vector linearized as described for pBAD33-g8-EcoRV construction (above) was assembled with a PCR product containing the entire Esp1396I RM system amplified from the pEsp1396IRM5.6 plasmid (4) (primers Esp F, Esp R). | https://benchling.com/s/seq-loM0Y1GY9JyHGPbBteOa?m=slm-OM2sAw9eaQ1HSqXNllBu |
| pBR322 | pMB1 ori; Tet^R^, Amp^R^ | Lab collection, (5) | https://benchling.com/s/seq-dRIlEXyK9aWBV6MvKmGt?m=slm-59ijluBGXArg9yCR4IO9 |
| pBR322-g8 | pBR322 with g8 protospacer with PAM | A linearized pBR322-g8 vector was prepared by inverse PCR (primers F-pBR322_lin, R-pBR322_lin) and assembled with a PCR product containing g8 protospacer with PAM region amplified from pBAD33-g8 (primers F-g8-pBR322, R-g8-pBR322). | https://benchling.com/s/seq-p4PTIFMsEM6VIduRHozz?m=slm-e92jH1zI0TTJ6NeCRuCE |
| pBR322-g8-Esp1396I | pBR322-g8 with the Esp1396I RM system | pBR322 was linearized using a restriction endonuclease EcoRI (Thermo Scientific) and assembled with a PCR product containing the entire Esp1396I RM system amplified from the pEsp1396IRM5.6 plasmid (4) (primers Esp_toBR_F, Esp_toBR_R). | https://benchling.com/s/seq-G18VvrkwVyo50DwYe9v7?m=slm-1N35jZbglxbZjdR2k6jW |
| pDualrep2 | KanR; Double reporter system containing *rfp* gene under *sulA* promoter and the *Katushka2S* gene downstream of the modified tryptophan attenuator under the strong constitutive T5 promoter. | (6) |  |

**Table S2. Primers used.**

| **Name** | **Sequence, 5’-3’** |
| --- | --- |
| F-g8-pBR322 | CGGGGTTGCCTTACTGGTTAGGCGGGATCGTCAC |
| R-g8-pBR322 | CGCGTATCGGTGATTCATTCTGATGCTGTCTTTCGCTG |
| F-pBR322_lin | CAGAATGAATCACCGATACGCGAG |
| R-pBR322_lin | CTAACCAGTAAGGCAACCCCGC |
| pBAD_EcoRV_GA_F | CGGGGATCCTCTAGAGTCGAC |
| pBAD_EcoRV_GA_R | TGACGATCAACTCTATTTCTCGCG |
| EcoRV_frag_F | CCGCGAGAAATAGAGTTGATCGTCAGCTGCAGGCATCGTGG |
| EcoRV_frag_R | GCAGGTCGACTCTAGAGGATCCCCGATCCTAGAGCGCACGAATGAGG |
| Eco29_F | CCGCGAGAAATAGAGTTGATCGTCAGAATCGCTCACGACTCG |
| Eco29_R | GCAGGTCGACTCTAGAGGATCCCCGCAACCCGGTAAGACACG |
| EcoRI_F | CCGCGAGAAATAGAGTTGATCGTCAGTTCGGTGGATTTTGACG |
| EcoRI_R | GCAGGTCGACTCTAGAGGATCCCCTTGTAATCGTTTGTTTTTTATC |
| Esp F | CCGCGAGAAATAGAGTTGATCGTCAAGAGTACTTGAAGTGGTGGC |
| Esp R | GCAGGTCGACTCTAGAGGATCCCCAGGAAGCATGTCACTCTCCG |
| Esp_toBR_F | AGCTGTCAAACATGAGAATTAGGAAGCATGTCACTCTCCG |
| Esp_toBR_R | CCCTTTCGTCTTCAAGAATTGAGTACTTGAAGTGGTGGCC |

**Fig. S1.** **The Esp1396I, EcoRI, Eco29kI, and EcoRV RM systems protect cells from λ_vir_ infection.**

Protection levels were determined by dividing the titers of λ_vir_ phage lysate on lawns of *E. coli* KD263 cells without plasmids by titers determined on lawns of KD263 cells harboring indicated plasmids. Means ± SE from triplicate experiments are shown.


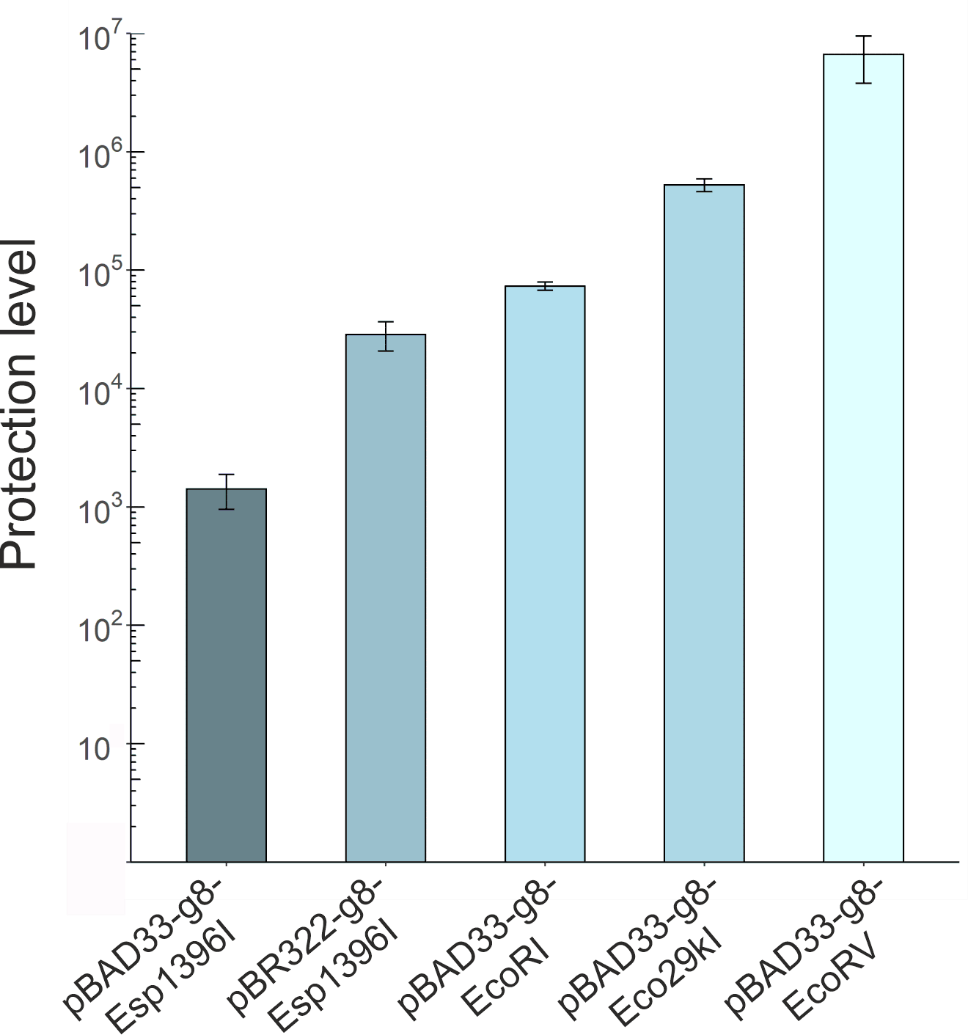


**Fig. S2**. **Plasmid loss induced by** **CRISPR interference.**

**(A)** The experimental setup of the assay is schematically shown (see also (7)). KD263 cells harboring g8 protospacer containing plasmids or control (no protospacer) plasmids were grown in LB without antibiotics with *cas* gene inducers or left uninduced. As cultivation was continued, culture aliquots were withdrawn each hour and plated on LB agar with and without antibiotics to estimate plasmid elimination rate. Plasmid DNA was extracted from culture aliquots and analyzed by agarose gel electrophoresis.

**(B-C)** Plots demonstrate the number of antibiotic-resistant CFUs in cultures transformed with (**B**) pBAD33 (left) and pBAD33-g8 (right) or (**C**) pBR322 (left) and pBR322-g8 (right), with (blue line) or without (black line) *cas* genes induction. Means ± SE of triplicate experiments are shown. Yields of plasmids (bands in agarose gel) purified from culture aliquots withdrawn at indicated timepoints are shown below each plot.


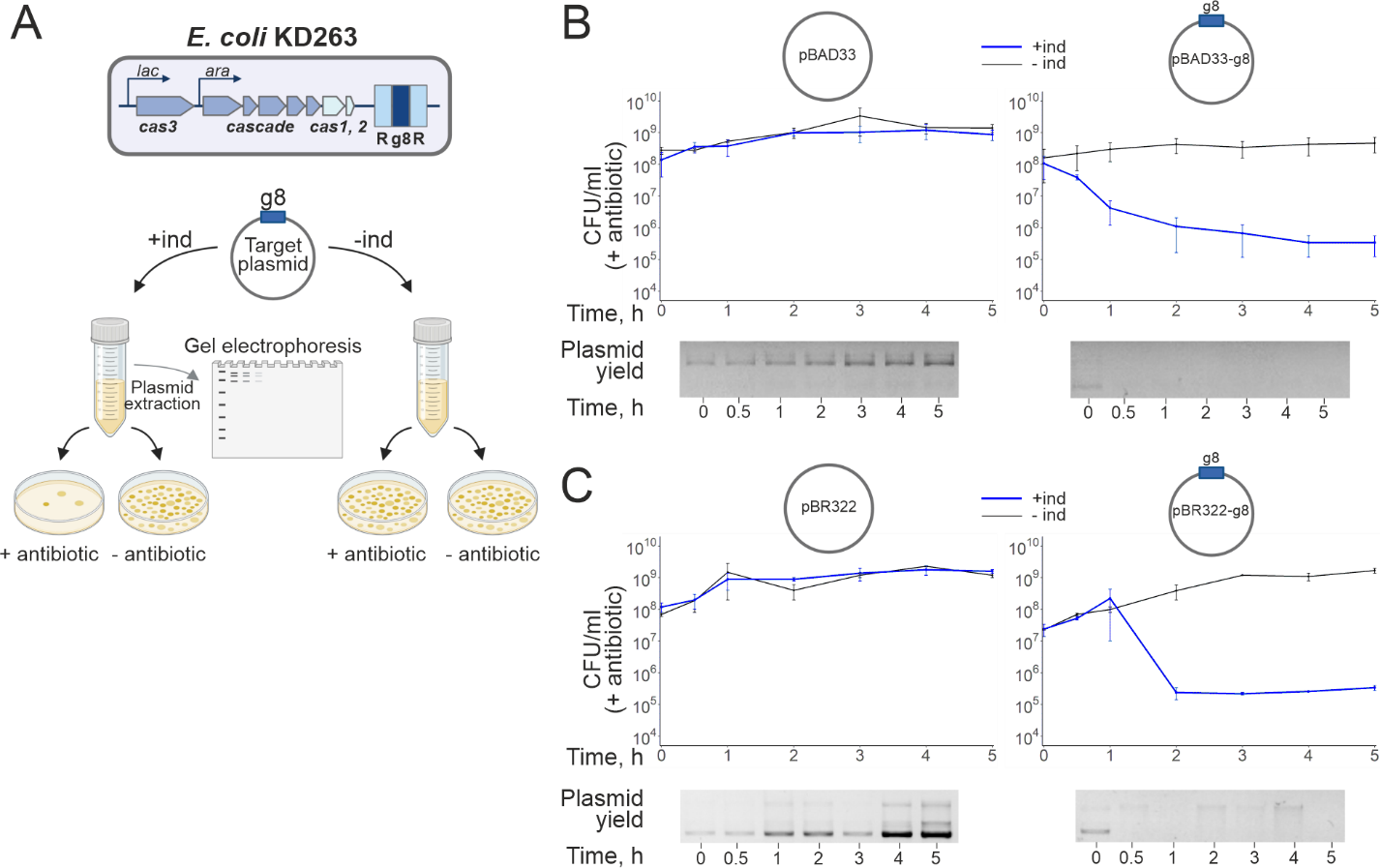


**Fig. S3. Quantification of cell divisions before elongation indicative of SOS response observed by microscopy.**

Percentages of cells divided once (green) or twice (orange) before elongated cells appear are shown. Number of cells used for calculation was >50 for each system.

**
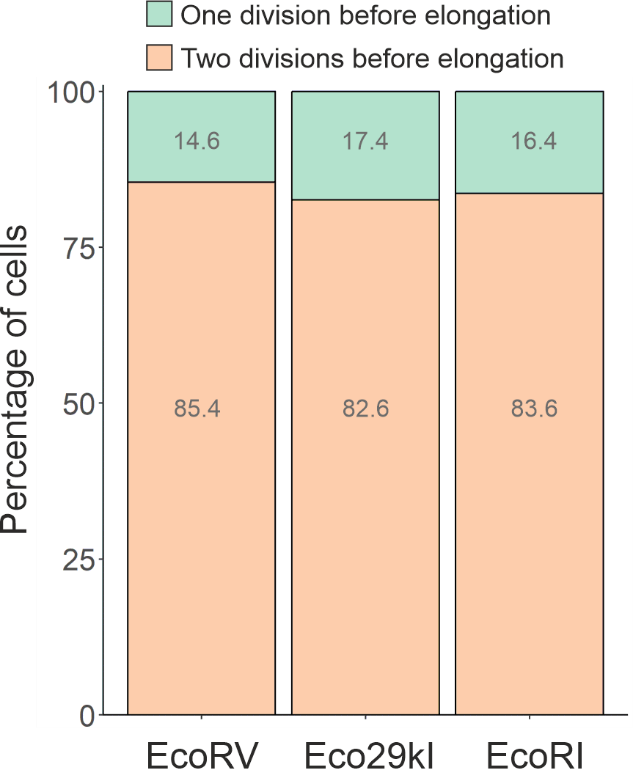
**

**Fig. S4. The growth of induced Esp1396I carrying cells is slower than the growth of control uninduced cells.**

**(A)** Time-lapse microscopy of growing Esp1396I carrying cells in the presence (bottom) and in the absence (top) of *cas* genes expression inducers. Time is shown in minutes.

**(B)** Number of cells in a field of view for uninduced (black rectangles) and induced (red circles) Esp1396I carrying cells.


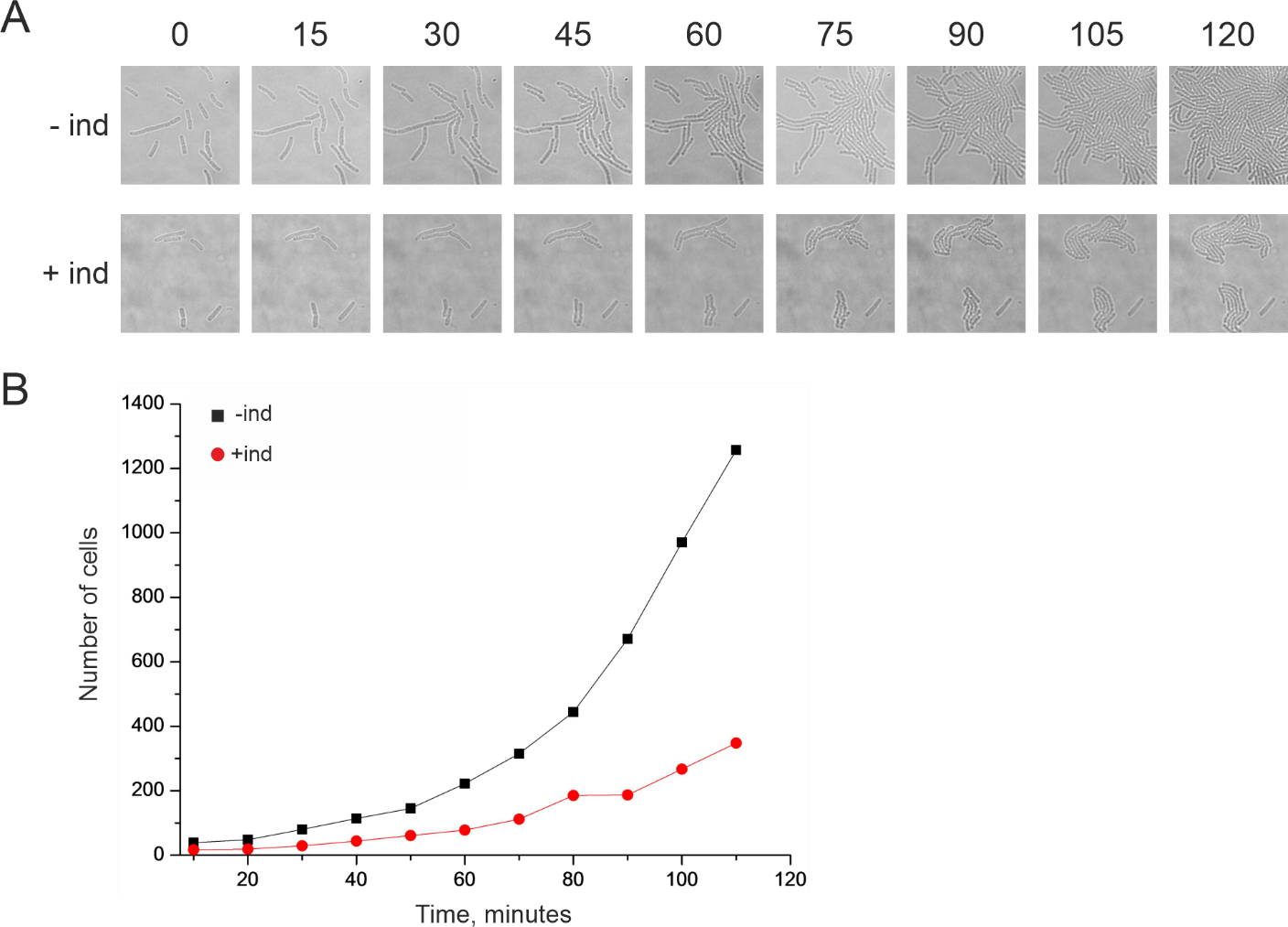


**Fig. S5. *ΔrecA* mutation in KD263 cells does not influence PSK for Esp1396I system.**

Plots demonstrate the number of colonies formed on LB plates without antibiotics. See Fig. 2 legend for details. Means ± SE of triplicate experiments are shown.


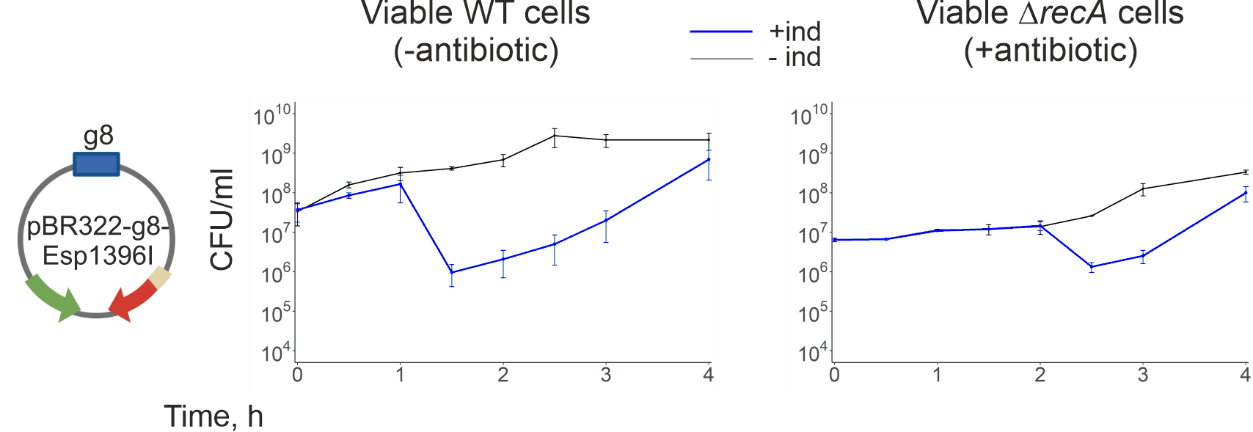


**Fig. S6. *In vitro* digestion on phage λ DNA in extracts of control cells or cells carrying plasmids with indicated RM systems.**

Cell-free extracts were combined with purified λ DNA and incubated for indicated times (in minutes). Digestion reactions were terminated by the addition of EDTA and samples were analyzed by agarose gel electrophoresis. On top panels, extracts were prepared from cells without any vector (top left) or carrying pBAD33-g8 vector (top right). On panels shown on the left, extracts were prepared from uninduced cells. On panels shown on the right, extracts were prepared from cells 2 hours after the induction of *cas* genes expression. Arrows indicate positions of digestion products specific for each REase.

**
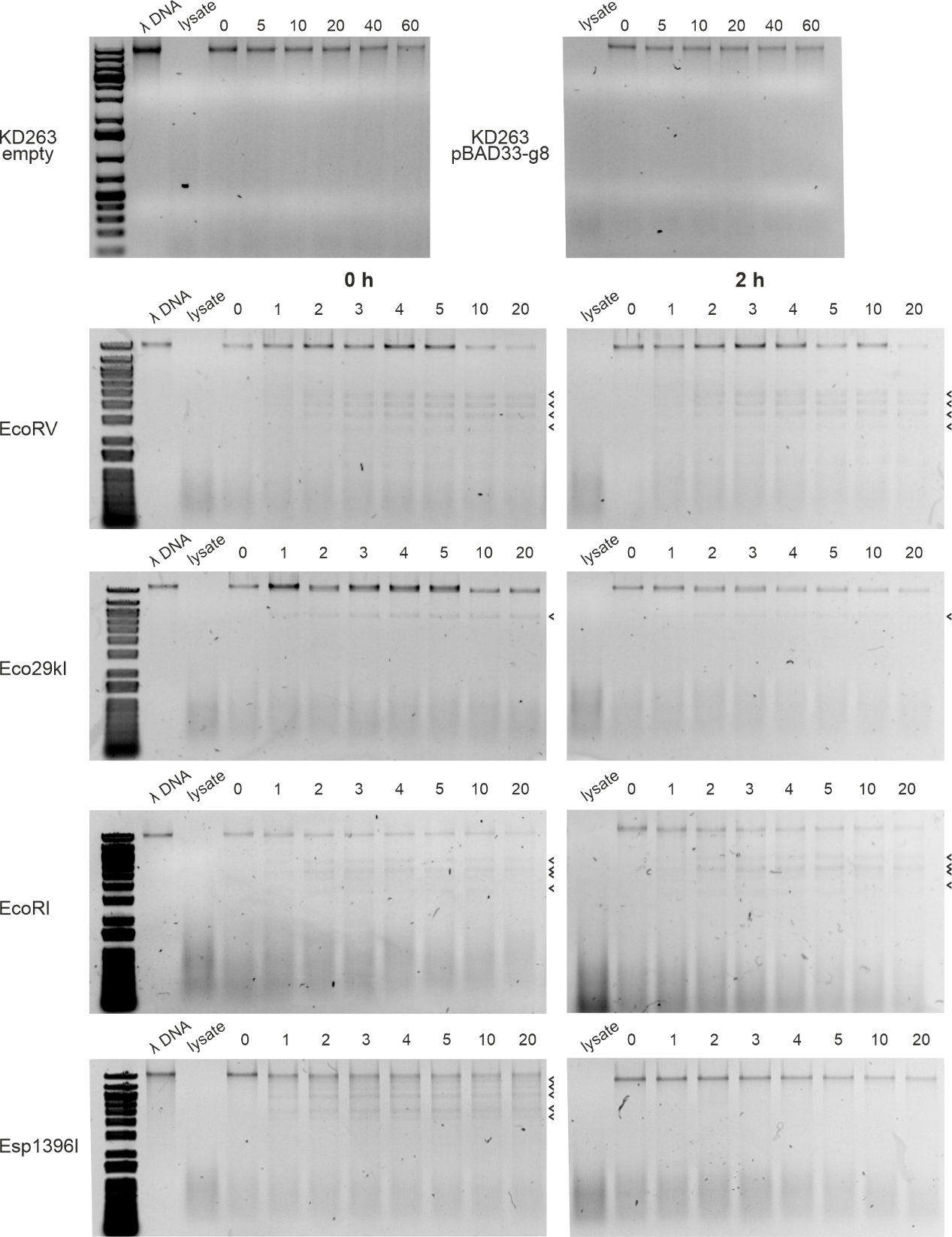
**

**Fig. S7. *In vitro* digestion on phage λ DNA in extracts of cells carrying the pBR322-g8-Esp1396I plasmid.**

Digestions was performed as described in Fig. 5 legend. On the top left panel, extract was prepared from uninduced cells. On other panels, extracts were prepared from cells at indicated times after the induction of *cas* genes expression.


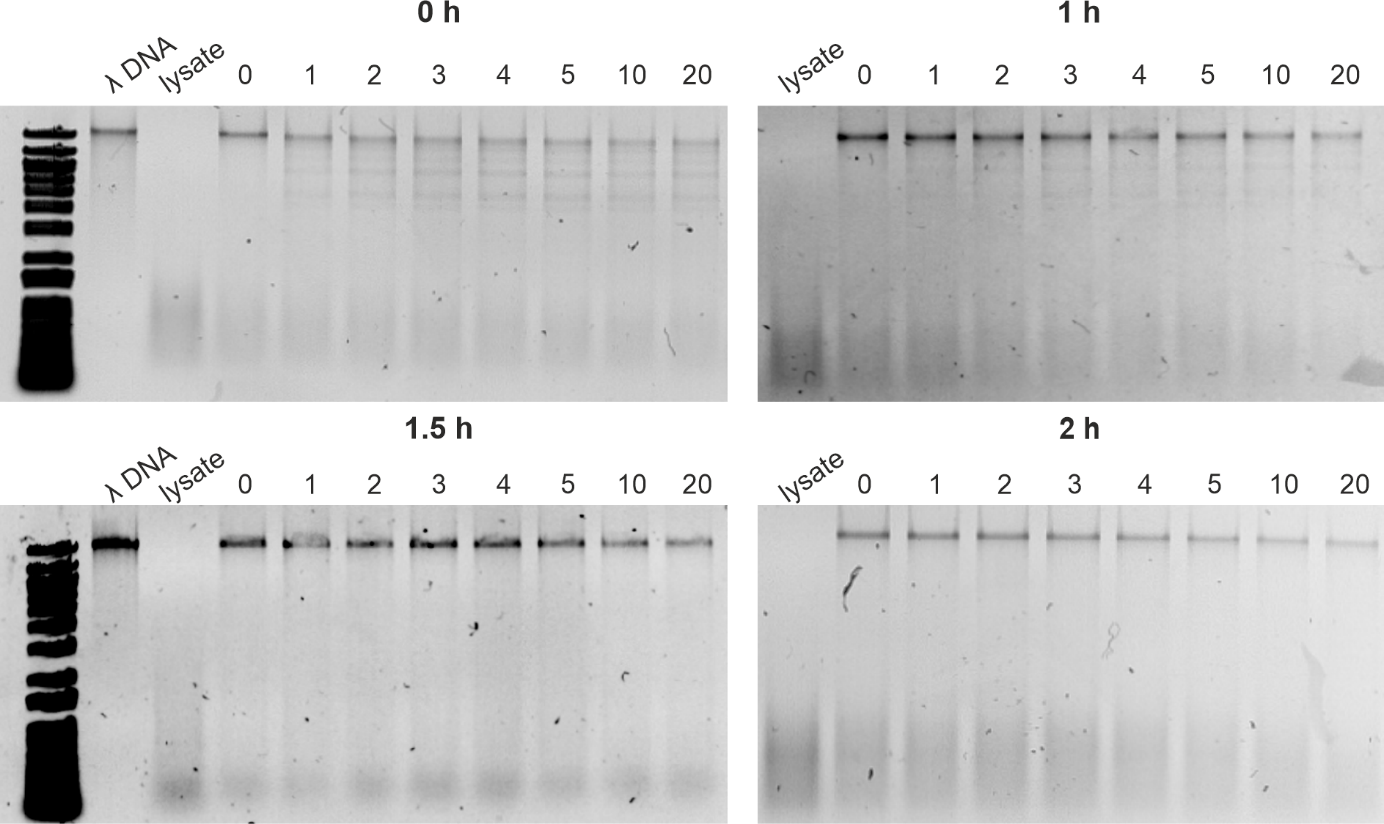


**References**

1. Guzman LM, Belin D, Carson MJ, Beckwith J. 1995. Tight regulation, modulation, and high-level expression by vectors containing the arabinose PBAD promoter. J Bacteriol 177:4121–4130.

2. Semenova E. 2005. Transcription regulation of the EcoRV restriction-modification system. Nucleic Acids Res 33:6942–6951.

3. Pertzev AV, Ruban NM, Zakharova MV, Beletzkaja IV, Petrov SI, Kravetz AN, Solonin AS. 1992. Eco29kI, a novel plasmid encoded restriction endonuclease from Escherichia coli. Nucleic Acids Res 20:1991.

4. Česnavičienė E, Mitkaitė G, Stankevičius K, Janulaitis A, Lubys A. 2003. Esp1396I restriction–modification system: structural organization and mode of regulation. Nucleic Acids Res 31:743–749.

5. Bolivar F, Rodriguez RL, Greene PJ, Betlach MC, Heyneker HL, Boyer HW, Crosa JH, Falkow S. 1977. Construction and characterization of new cloning vehicles. II. A multipurpose cloning system. Gene 2:95–113.

6. Osterman IA, Komarova ES, Shiryaev DI, Korniltsev IA, Khven IM, Lukyanov DA, Tashlitsky VN, Serebryakova MV, Efremenkova OV, Ivanenkov YA, Bogdanov AA, Sergiev PV, Dontsova OA. 2016. Sorting Out Antibiotics’ Mechanisms of Action: a Double Fluorescent Protein Reporter for High-Throughput Screening of Ribosome and DNA Biosynthesis Inhibitors. Antimicrob Agents Chemother 60:7481–7489.

7. Semenova E, Savitskaya E, Musharova O, Strotskaya A, Vorontsova D, Datsenko KA, Logacheva MD, Severinov K. 2016. Highly efficient primed spacer acquisition from targets destroyed by the *Escherichia coli* type I-E CRISPR-Cas interfering complex. Proc Natl Acad Sci 113:7626–7631.
